# Crystal structures of p120RasGAP N-terminal SH2 domain in its apo form and in complex with a p190RhoGAP phosphotyrosine peptide

**DOI:** 10.1371/journal.pone.0226113

**Published:** 2019-12-31

**Authors:** Rachel Jaber Chehayeb, Amy L. Stiegler, Titus J. Boggon

**Affiliations:** 1 Yale College, New Haven, Connecticut, United States of America; 2 Department of Molecular Biophysics and Biochemistry, Yale University, New Haven, Connecticut, United States of America; 3 Department of Pharmacology, Yale University, New Haven, Connecticut, United States of America; 4 Yale Cancer Center, Yale University, New Haven, Connecticut, United States of America; Universitetet i Bergen, NORWAY

## Abstract

The Rho and Ras pathways play vital roles in cell growth, division and motility. Cross-talk between the pathways amplifies their roles in cell proliferation and motility and its dysregulation is involved in disease pathogenesis. One important interaction for cross-talk occurs between p120RasGAP (*RASA1*), a GTPase activating protein (GAP) for Ras, and p190RhoGAP (p190RhoGAP-A, *ARHGAP35*), a GAP for Rho. The binding of these proteins is primarily mediated by two SH2 domains within p120RasGAP engaging phosphorylated tyrosines of p190RhoGAP, of which the best studied is pTyr-1105. To better understand the interaction between p120RasGAP and p190RhoGAP, we determined the 1.75 Å X-ray crystal structure of the N-terminal SH2 domain of p120RasGAP in the unliganded form, and its 1.6 Å co-crystal structure in complex with a synthesized phosphotyrosine peptide, EEENI(p-Tyr)SVPHDST, corresponding to residues 1100–1112 of p190RhoGAP. We find that the N-terminal SH2 domain of p120RhoGAP has the characteristic SH2 fold encompassing a central beta-sheet flanked by two alpha-helices, and that peptide binding stabilizes specific conformations of the βE-βF loop and arginine residues R212 and R231. Site-directed mutagenesis and native gel shifts confirm phosphotyrosine binding through the conserved FLVR motif arginine residue R207, and isothermal titration calorimetry finds a dissociation constant of 0.3 ± 0.1 μM between the phosphopeptide and SH2 domain. These results demonstrate that the major interaction between two important GAP proteins, p120RasGAP and p190RhoGAP, is mediated by a canonical SH2-pTyr interaction.

## Introduction

The Ras pathway is involved cell proliferation, differentiation, migration and apoptosis [1], and the Rho GTPases are essential in cell adhesion, protrusion, polarity, migration and cell motility [2]. These pathways interact with one-another, in what is known as ‘cross-talk’ [3–6], but the exact basis for Ras-Rho cross-talk is not fully understood, and represents a deficiency in the understanding of how these pathways function. One of the ways by which Rho and Ras pathways are thought to interact is by the direct binding of two GTPase-activating proteins (GAPs), p190RhoGAP and p120RasGAP (*RASA1*) [7–9].

**Fig 1:**
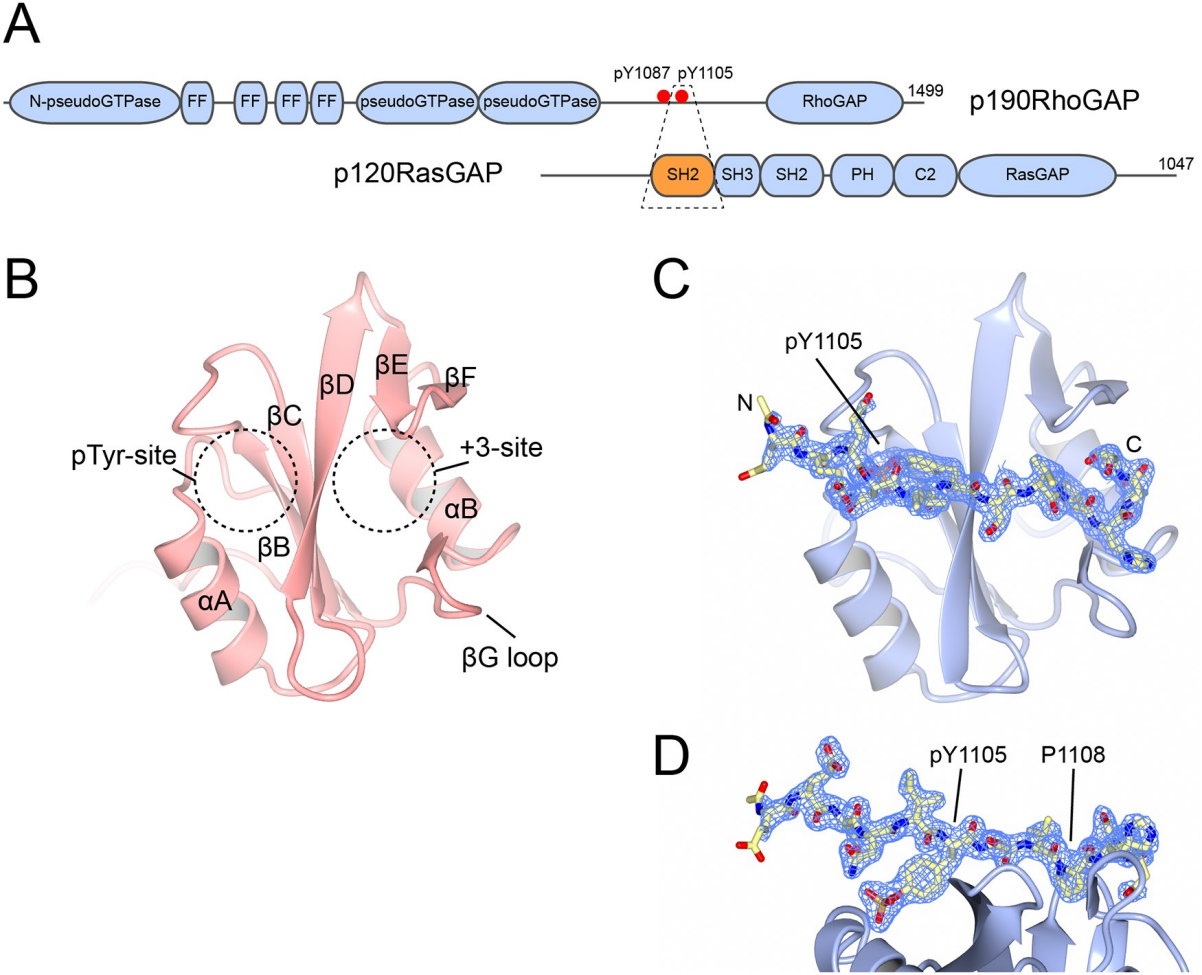
Structure of apo p120RasGAP SH2 domain and its complex with p190RhoGAP pTyr-1105 peptide. **A)** Domain organizations of p190RhoGAP-A and p120RasGAP. The region that is co-crystallized is highlighted by a dashed box. Domains are indicated: FF, FF domain; SH2, Src-homology 2; SH3, Src-homology 3; PH, pleckstrin homology; C2, C2 domain. C-terminal residue number labeled. **B)** Overall structure of the N-terminal SH2 domain of p120RasGAP. Secondary structure elements are indicated. Locations of the pTyr binding site, and the specificity determining +3 site are indicated. **C)** Overall structure of the N-terminal SH2 domain of p120RasGAP in complex with a peptide corresponding to residues 1100–1112 of p120RasGAP. p120RasGAP is shown in cartoon format. p190RhoGAP is shown in stick format. pTyr-1105 is indicated. 2*F*_obs_-*F*_calc_ electron density for the p190RhoGAP peptide is shown at a contour level of 1σ in blue. Positions of peptide residues are indicated. **D)** Side view of C, highlighting positions and density of pTyr-1105 and Pro-1108.

p190RhoGAP is a multidomain RhoGAP, and the two isoforms (-A and -B; *ARHGAP35* and *ARHGAP5*, respectively) are significant regulators of Rho signaling [7, 10, 11]. The proteins contain multiple domains, including N-terminal pseudoGTPase domains [12–14], and a C-terminal RhoGAP [15–17] (Fig 1A). Between these domains there is a flexible region that has been found to be tyrosine phosphorylated; either one or both of the phosphotyrosine sites at residues Tyr-1087 and Tyr-1105 are responsible for p190RhoGAP’s recruitment to the plasma membrane where appropriate regulation of Rho cascades is achieved [9, 18–21]. Src, Abl2 and other tyrosine kinases have been shown to phosphorylate Tyr-1105 [7–9], and this site is the most frequently observed [22], and is critical for normal p190RhoGAP function, including its direct interaction with p120RasGAP at the plasma membrane [3, 18, 19].

p120RasGAP is a multidomain regulator of Ras signaling, containing two SH2 domains at its N-terminus and multiple other domains including a RasGAP [23–25] (Fig 1A). Binding of p190RhoGAP is mediated by phosphotyrosine-SH2 domain interactions, and a major driver is thought to be between the N-terminal SH2 of p120RasGAP and pTyr-1105 of p190RhoGAP [18, 19]. p190RhoGAP recruitment has multiple effects, including inhibition of Rho signaling by locating p190RhoGAP to the plasma membrane [26–30], and activation of Ras signaling by suppression of p120RasGAP’s RasGAP activity when it is bound to p190RhoGAP [3, 31].

p120RasGAP was the first GAP to be identified [24, 32–34], is a major downregulator of RAS signaling [35–39], and is extensively mutated in the *RAS*opathy, capillary malformation-arteriovenous malformation (CM-AVM) [40–43], however, the structural basis of its signaling is not well understood. Likewise, the interaction of p120RasGAP and p190RhoGAP was first identified in 1995 [7], but further molecular level studies have not been pursued. Therefore, in this study we determine the crystal structures of the N-terminal SH2 domain of p120RasGAP alone, and its complex with a phosphorylated peptide corresponding to residues around and including pTyr-1105 of p190RhoGAP. These crystal structures demonstrate a canonical SH2-pTyr interaction between the proteins and provides the first structural study defining the interaction of p190RhoGAP and p120RasGAP.

## Materials and methods

### Expression and purification of WT and R207A mutant p120RhoGAP N-SH2 domain

cDNA of residues 174 through 280 of human p120RasGAP (UniProt ID: P20936) encoding the N-terminal SH2 domain tagged was subcloned into a modified pET28a vector with an N-terminal hexahistidine tag and TEV protease recognition sequence. Two native cysteines, C236 and C261, were mutated to serine to prevent disulfide bridge formation. Following transformation into Rosetta(DE3) cells, a 1L culture was induced overnight at OD_600_ of 0.6–0.8 and 18°C using 0.2 mM IPTG. Cells were harvested by centrifugation and resuspended in lysis buffer (50 mM HEPES pH 7.3, 500 mM NaCl). Lysis was facilitated by lysozyme addition and three freeze/thaw cycles, prior to sonication and DNAse I addition. Clarified lysate was prepared by centrifugation at 5000 x *g* at 4°C and incubated with nickel beads (Ni-NTA Agarose, Qiagen) for 1 hr at 4°C to capture hexahistidine-tagged protein. Following washing with 3 column volumes of wash buffer (50 mM HEPES pH 7.3, 500 mM NaCl, 20mM imidazole), TEV protease was added overnight at 4 °C to cleave the SH2 domain protein from the hexahistidine-tag. The next day, flow through containing tag-free SH2 domain protein was collected and the beads were washed with several column volumes of wash buffer to fully remove the cleaved protein from the beads. Size exclusion chromatography (Superdex 75, GE Healthcare) was performed on p120RasGAP N-SH2 in 20 mM Tris pH 7.4 and 150 mM NaCl. Yield was 20 mg/L culture. Protein was concentrated by centrifugal filter (Amicon Ultra, Millipore Sigma).

A mutant form, with R207A mutation, was introduced by QuikChange mutagenesis (Agilent) using forward and reverse primers (5'-tccgatcactctctgctataagataactgccagacttccct-3', 5'- agggaagtctggcagttatcttatagcagagagtgatcgga-3’). Expression and purification were by identical methods as wild type protein. Yield for the mutant was 4 mg/L culture. Both wild type and mutant constructs elute from size exclusion chromatography as monodisperse peaks.

### Peptide synthesis

A synthetic 13 amino acid peptide of sequence EEENI(p-Tyr)SVPHDST native to p190RhoGAP residues 1100 to 1112 phosphorylated at Tyr-1105, with N-terminal acetylation and C-terminal amidation, was commercially synthesized (GenScript) and re-suspended in sterile-filtered water.

### Crystallization and data collection

The purified N-terminal SH2 domain of p120RasGAP was concentrated to 17.5 mg/mL and initial crystal screening conducted using Index HT and PEG Rx HT (Hampton Research). Hits were observed in precipitant conditions of 0.2 M ammonium acetate, 0.1 M Tris pH 8.0 and 16% w/v polyethylene glycol 10,000 and grid screening conducted using hanging drop vapor diffusion VDX plates with 1 μL:1 μL protein: reservoir solution ratio suspended over 500 μL reservoir solution. Optimized crystals grew against 0.2 M ammonium acetate, 0.1 M Tris pH 8.0 and 21% w/v PEG 10,000 at room temperature. For data collection, crystals were cryoprotected in precipitant solution containing 34% w/v glycerol and flash-frozen in liquid nitrogen.

For co-crystallization of wild-type protein with phosphopeptide, native PAGE was first conducted to determine saturating phosphopeptide concentration. The protein-peptide mixtures used throughout the screening and optimization process had a saturating 1.7:1 peptide:protein molar ratio. Initial screening was conducted using Index HT, PEG Rx HT and PEG Ion HT screens (Hampton Research). Initial crystal hits were observed in conditions containing 1.8 M ammonium sulfate, 0.1 M Bis-Tris pH 6.5, 2% PEG MME 550 yielding 2-dimensional needle cluster crystals. Following optimization, single crystals grew against 1.8 M sodium malonate, 0.1 M Bis-Tris pH 6.5, 2% PEG MME 550 at room temperature using hanging drop vapor diffusion VDX plates with a 1 μL:1 μL ratio of a pre-mixed protein-peptide solution to reservoir solution suspended over 500 μL reservoir solution. Crystals were cryo-protected in reservoir buffer containing 2.9 M sodium malonate and flash-frozen in liquid nitrogen.

### Structure determination and refinement

X-ray diffraction data for both crystals were collected at the Northeastern Collaborative Access Team (NE-CAT) beamline 24-ID-C at Argonne National Laboratory and reduced using HKL2000 [44]. For the apo structure, data were processed to 1.75 Å resolution. Initial scaling and data quality assessment in Phenix Xtriage [45] supported a spacegroup of *P*3_2_12 and detected the presence of translational pseudosymmetry with an off-origin Patterson function peak at height of 71.5%. Molecular replacement in *P*3_2_12 by Phaser [46] using the C-terminal SH2 domain of phospholipase C-γ (PDB ID: 4K44) as a search model yielded a solution containing three copies with a translation function Z-score (TFZ) of 9.3; however, Phenix autobuild [47] and refinement stalled at *R*_free_ values around 50%. Thus, Zanuda [48] was used to aid in correct spacegroup assignment of *P*3_2_ with 6 copies of SH2 per asymmetric unit. Reflection data were then reprocessed in *P*3_2_ in HKL2000 [44]. Molecular replacement was performed in Phaser [46] using the C-terminal SH2 domain of phospholipase C-γ (PDB ID: 4K44) as a search model, and six copies were placed with a TFZ score of 12.2. Phenix autobuild [47] built 580 residues. For the peptide-bound structure, Matthews analysis predicted one copy in the asymmetric unit. Molecular replacement by Phaser [46] using the C-terminal SH2 domain of phospholipase C-γ (PDB ID: 4K44) as a search model yielded a single solution with a translation function Z-score (TFZ) of 11.3. Phenix autobuild [47] built 92 residues of the p120RhoGAP N-SH2 domain and 8 residues of the p190RhoGAP peptide. Residues of the peptide were updated to the correct sequence, and manual building conducted using Coot [49].

For both structures, multiple rounds of manual model building using Coot [49] and refinement using Phenix [45] were conducted, with NCS applied in the apo structure refinement. The density for the p190RhoGAP peptide is clear, and MolProbity [50] found good geometry for the final models. The two introduced cysteine to serine mutations, C236S and C261S, lie within linker loops and do not impact secondary structure. The asymmetric unit of the apo structure is composed of three dimers which we attribute to crystal packing: chains A/B, C/D, and E/F. Final *R* and *R*_free_ values for the apo structure are 18.5% and 21.2%, respectively. For the peptide-bound structure the final *R* and *R*_free_ values are 22.0% and 26.3%, respectively (Table 1). Structural figures were generated using CCP4mg [51]. The PISA server was used for analysis of the protein-peptide interaction interface [52]. All crystallography software was compiled by SBGrid [53].

**Table 1:** Data collection and refinement statistics.

Data Collection	Apo	Peptide Bound
PDB accession code	6PXB	6PXC
Wavelength (Å)	0.97920	0.97910
Resolution range (Å)	50–1.75 (1.81–1.75)	50–1.6 (1.66–1.6)
Space group	*P* 3_2_	*I* 2 2 2
Cell dimensions		
a, b, c (Å)	64.3, 64.3, 119.4	44.2,64.8, 87.2
α, β, γ (°)	90, 90, 120	90, 90, 90
Unique reflections	55502 (5568)	16868 (1652)
Multiplicity	9.9 (8.0)	15.8 (8.6)
Completeness (%)	99.9 (99.9)	100 (99.9)
Mean *I*/σ*I*	29.1 (1.8)	17.7 (2.6)
Wilson B factor (Å^2^)	28.3	28.8
*R*_pim_ (%)	2.6 (30.3)	3.9 (39.8)
CC½	1.01 (0.91)	1.01 (0.73)
CC*	1.00 (0.98)	1.00 (0.92)
**Refinement**		
Resolution range (Å)	40.6–1.75 (1.8–1.75)	43.6–1.60 (1.7–1.6)
Reflections used in refinement	55130 (2617)	16849 (2549)
Reflections used for *R*_free_	2810 (138)	845 (138)
*R*_work_ (%)	22.0 (37.5)	18.5 (26.7)
*R*_free_ (%)	26.3 (39.8)	21.0 (28.9)
No. of non-hydrogen atoms	5192	1005
SH2 domain	4920	841
Peptide	0	106
Solvent	272	56
Protein residues	611	115 (104 SH2, 13 peptide)
RMSD		
Bond lengths (Å)	0.013	0.007
Bond angles (°)	1.388	0.893
Ramachandran plot (%)		
Favored, allowed, outliers	98.7, 1.3, 0	98.2, 1.8, 0
Rotamer outliers	5.8	2.0
MolProbity clashscore	11.1 (62^nd^ percentile)	2.7 (99^th^ percentile)
Average B factor (Å^2^)	50.7	44.7
SH2 domains	50.9	44.1
Copies		
A, B, C	47.7, 46.4, 51.6	43.12
D, E, F	57.7, 52.4, 50.9	
Peptide	-	51.6
Solvent	47.7	51.4

Statistics for the highest-resolution shell are shown in parentheses. RMSD, root-mean-square deviation.

### Native polyacrylamide gel electrophoresis (PAGE)

A serial dilution series of pTyr-1105 phosphopeptide was performed in dilution buffer (50 mM Tris pH 7.4), and mixed with wild-type or R207A mutant N-terminal SH2 domain protein for a final protein concentration of 0.08 mM and a phosphopeptide concentration ranging from 0.02 mM to 0.15 mM (peptide:protein ratio ranging from 0.25 to 1.9). The protein-peptide mixtures, or protein alone as negative control, were incubated on ice for 10 minutes and then centrifuged for 1 minute at 4 °C. 10 μL of 2X native protein sample buffer (62.5 mM Tris HCL pH 6.8, 40% glycerol, 0.01% bromophenol blue) was then added to all samples. 15 μL of each sample (containing 10 μg) was loaded onto a 4–20% Mini-PROTEAN TGX Stain-Free Precast gel (Bio-Rad), and proteins resolved in native gel buffer (25 mM Tris, 192 mM glycine, pH 8.3) at 200 V for 1 hour at room temperature. Protein bands were visualized by staining with Coomassie Brilliant Blue R 250 stain.

### Isothermal titration calorimetry (ITC)

The N-SH2 protein and pTyr1105 peptide were prepared for ITC by overnight dialysis in a common buffer with a 20mM Tris 7.4, 150mM NaCl composition. Slide-A-Lyzer Dialysis Cassettes with a molecular weight cut-off of 3,500 Da were used for protein dialysis and Micro Float-A-Lyzer Dialysis devices with a 100–500 Da cut-off for peptide dialysis. Samples were retrieved from their cartridges, spun down for 10 minutes at 4°C. Concentrations were measured using a Nanodrop spectrophotometer at 280 nm. To determine peptide concentration, a phosphotyrosine extinction coefficient of 458.6 M^-1^cm^-1^ at pH 7.4 was used [54]. Two ITC experiments were performed using a Nano-ITC (TA Instruments). Both protein and peptide were degassed for 3 minutes prior to loading. 350 μL of protein were injected into the sample cell. The injection burette was loaded with 52 μL of peptide. 20 injections of 2.5 μL of peptide, each spanning 300 seconds, were performed per ITC run. Results were analyzed using the nano-ITC software, through fitting to an independent binding model. Observed values for two runs were: K_a_ = 2.3 x10^-5^ M^-1^, N = 0.724, ΔH = -17.6 kcal/mol, K_d_ = 0.43 μM, TΔS = -8.9 kcal/mol, ΔG = -8.7 kcal/mol; and K_a_ = 4.2 x10^-6^ M^-1^, N = 0.63, ΔH = -13.9 kcal/mol, K_d_ = 0.24 μM, TΔS = -4.9 kcal/mol, ΔG = -9.0 kcal/mol.

## Results and discussion

### Crystal structures of apo and peptide-bound p120RasGAP N-terminal SH2 domain

Direct interaction of the N-terminal SH2 domain of p120RasGAP and pTyr-1105 of p190RhoGAP in large part mediates the direct interaction of these proteins [18, 19]. The molecular basis for the interaction has not, however, been described. We therefore determined the crystal structure of this SH2 domain alone and in complex with a peptide corresponding to the pTyr-1105 region. We began by expressing and purifying the N-terminal SH2 domain of p120RasGAP alone, and by obtaining apo crystals of this domain. These crystals diffracted well, and we obtained a 1.75 Å dataset that contains 6 copies per asymmetric unit. Of the 6 copies, chains A and B show significantly lower *B*-factors and improved electron density compared to the other copies and we have based our analyses on these chains. We next obtained co-crystals of the N-terminal SH2 domain of p120RasGAP in complex with a synthesized peptide, EEENI(p-Tyr)SVPHDST, corresponding to residues 1100 to 1112 of p190RhoGAP. The co-crystals diffracted to 1.6 Å resolution and contain one dimer per asymmetric unit. Good electron density is observed for all residues of the peptide except Thr-1112. The crystal structures of the N-terminal p120RasGAP SH2 domain and the p120RasGAP-p190RhoGAP complex are the first for the N-terminal SH2 domain, and the first showing interaction of p190RhoGAP and p120RasGAP.

### Overall structure of the p120RasGAP N-terminal SH2 domain

The SH2 fold consists of a central β-sheet flanked by two α-helices [55–59], and both crystal structures reveal that the p120RasGAP N-terminal SH2 domain adopts this fold. Following the naming conventions, there are two alpha helices, αA and αB, which sandwich an antiparallel beta sheet (strands βB, βC and βD) that is extended by two short β-strands, βE and βF (Fig 1B). In canonical SH2 domains, peptide binding occurs perpendicular to the β-sheet, with the phosphotyrosine (position 0) binding site located between the β-sheet and helix αA. The specificity determining +3 position (in Src this is the isoleucine of the preferred -pY-E-E-I- binding site) is located on the opposite side of the β-sheet, flanked by helix αB, and by the βE-βF loop and the βG loop immediately C-terminal to helix αB (Fig 1B). The interaction between p120RasGAP SH2 domain and the p190RhoGAP pTyr-1105 peptide follows this convention and is supported by good electron density (Fig 1C and 1D).

### Conformational sampling of the apo SH2 domain of p120RasGAP

**Fig 2:**
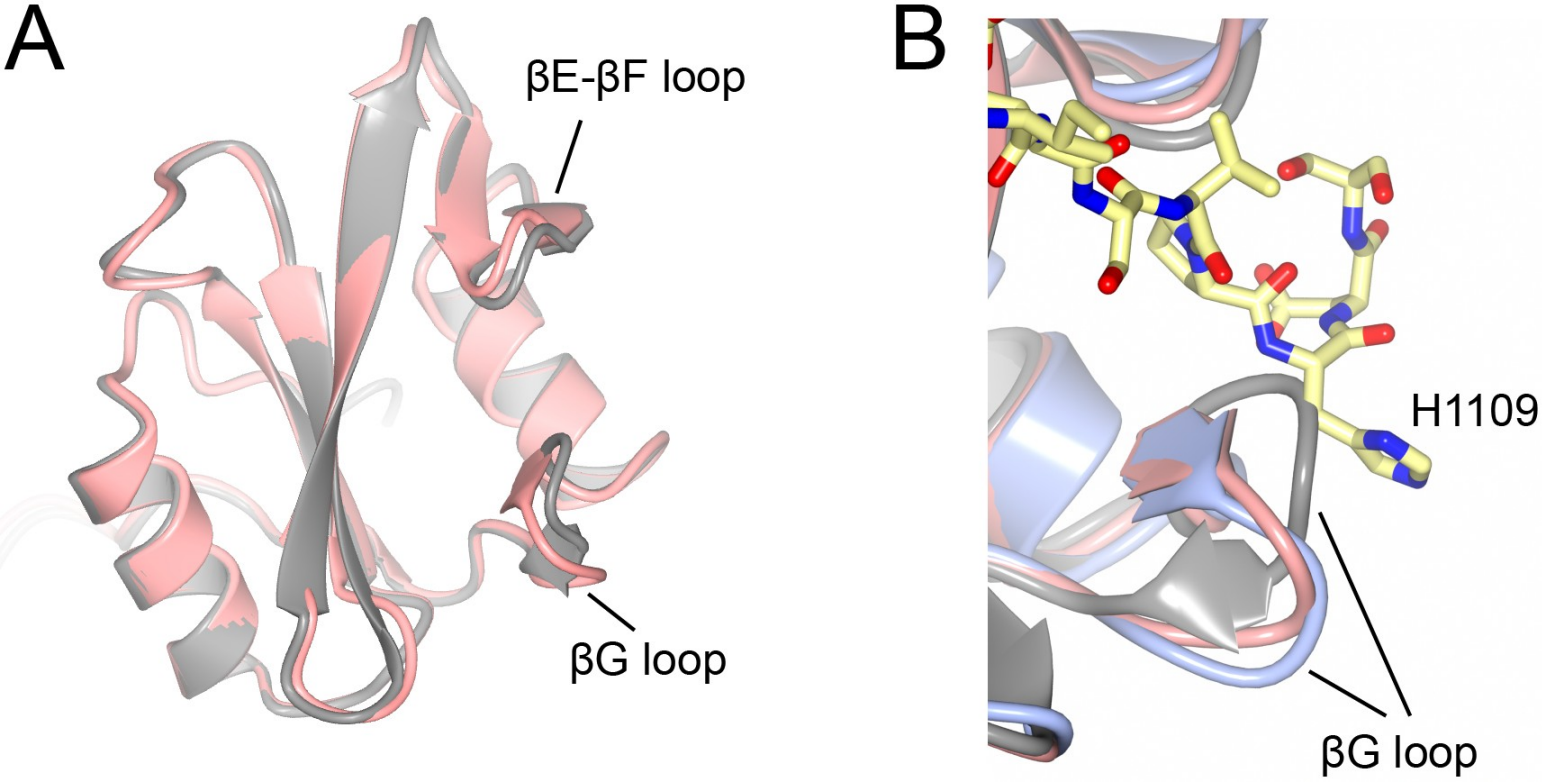
Conformational sampling of the apo structures of p120RasGAP N-SH2. **A)** Conformational differences between the poised (pink) and occluded (grey) states observed in the asymmetric unit of the crystal structure. **B)** Superposition of the poised and occluded states onto the complexed structure and indicates that the occluded conformation clashes with peptide residue His-1109.

In our crystal structure of apo p120RasGAP, we observe six copies per asymmetric unit arranged as dimers (chains A/B, C/D, E/F). Superposition of the crystallographic dimers reveals that there are two conformational classes, which can be defined by the conformation of the βG and βE-βF loops. Interestingly, these loops important for specificity determination at the +3 position of the phosphopeptide [60–62]. We observe that in one class the loops are open and poised for peptide binding, and in the other they are closer and seem to occlude peptide binding (Fig 2A). In both classes, the *B-*factors of βG loop are high, and in the occluded form this loop cannot be built in two of the three copies, however, when the bound phosphopeptide structure is superposed on these conformations the occluded state clashes with His-1109 at peptide position +4, but the poised conformation can accommodate binding (Fig 2B). We interpret this to indicate conformational flexibility and sampling of the specificity defining loops in the absence of phosphopeptide binding.

### Structural basis of p120RasGAP N-SH2 interaction with p190RhoGAP pTyr-1105

**Fig 3:**
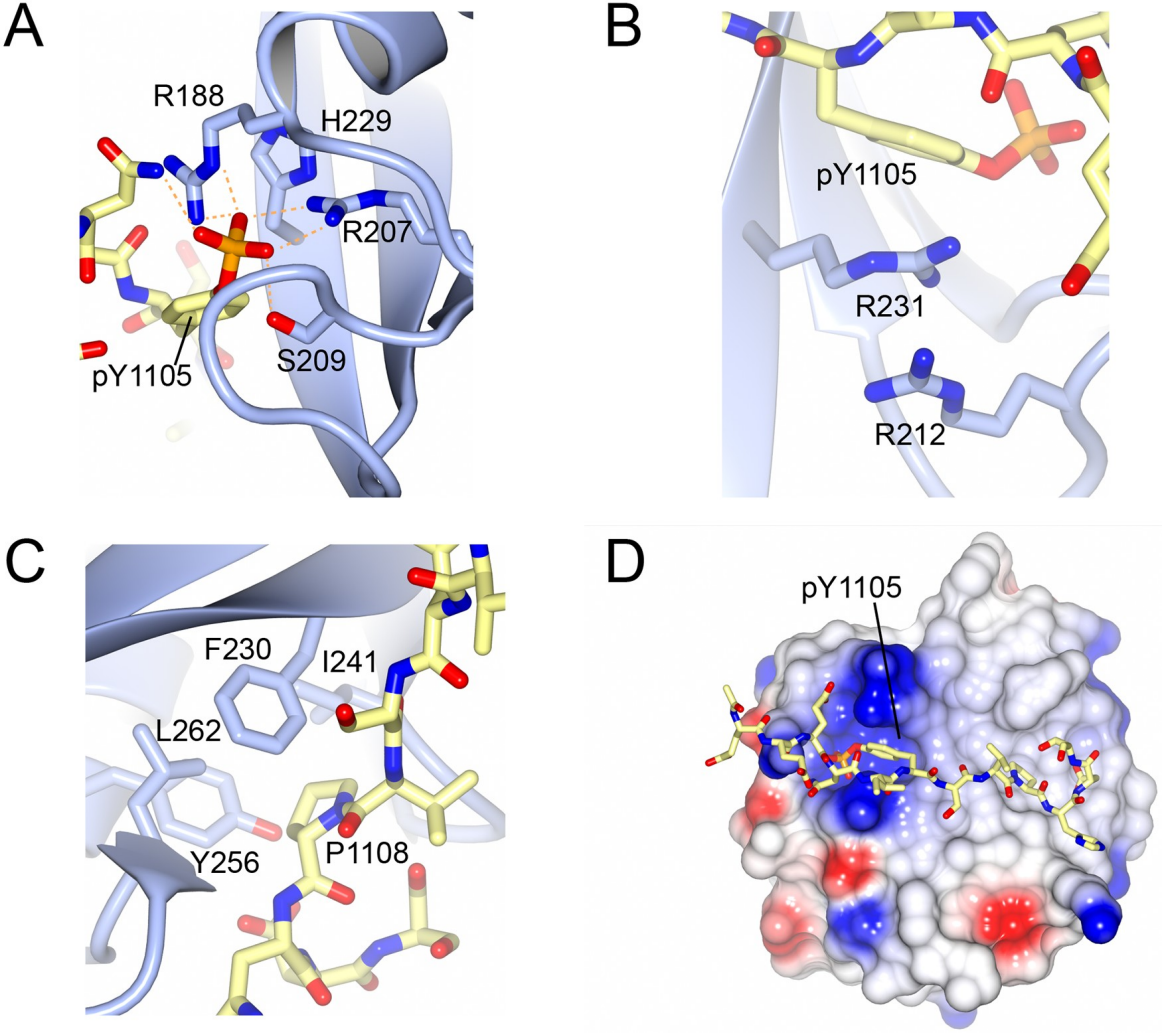
Features of the p120RasGAP-p190RhoGAP co-crystal structure. **A)** Close-up of the interactions between p190RhoGAP pTyr-1105 (yellow, stick) and p120RasGAP SH2 domain (blue, cartoon and stick). FLVR motif arginine is Arg-207. H-bonds are shown in orange. **B)** Cation-π stack between pTyr-1105, Arg-231 and Arg-212. **C)** Close-up of the +3 pocket. **D)** Surface electrostatics of p120RasGAP. p190RhoGAP shown in stick format.

The co-crystal structure reveals a broadly canonical interaction between p120RasGAP N-terminal SH2 domain and the p190RhoGAP pTyr-1105 peptide. The interaction buries 1290 Å^2^ of total surface area and the peptide binds perpendicular to the central SH2 domain β-sheet, with pTyr-1105 at position 0 and Pro-1108 at position +3 inserted into the expected pockets (Fig 1C). pTyr-1105 is coordinated by a salt-bridge to Arg-207, which is the conserved arginine of the FLVR motif [63], also commonly referred to as ArgβB5 in SH2 convention [56]. It is also coordinated by Arg-188 (ArgαA2) and Ser-209 (SerβB7) (Fig 3A). Interestingly, a three-residue cation-π stack is also observed between the phenyl-ring of pTyr-1105, Arg-231 and Arg-212 (Fig 3B). In the apo structure, we do not observe a similar orientation of arginines, Arg-231 and Arg-212, we therefore infer the cation-π stack to be induced by peptide binding.

Pro-1108 of p190RhoGAP is inserted into the specificity determining SH2 hydrophobic pocket between βG and βE-βF loops. The pocket is defined by residues Phe-230, Leu-262, Ile-241 and Tyr-256 (Fig 3C). Compared to Src, this is a shallow +3 specificity site and is thus predisposed for specificity towards proline, as has been shown experimentally [61]. In the co-crystal structure, the βE-βF loop is stabilized by peptide binding which allows a hydrogen-bond to form between the carbonyl oxygen of Leu-262 and the backbone nitrogen of His-1109. The βE-βF loop displays lower relative *B*-factors compared to the apo structure (Fig 2B) and we interpret this to indicate stabilization of the βE-βF loop upon peptide binding. Additional direct hydrogen bonds are also observed between the backbone carbonyl of Glu-1101 and Arg-211, between the backbone nitrogen of pTyr-1105 and the backbone carbonyl of His-229, and between Asp-1110 and both Tyr-256 and Ser-260 of p120RasGAP.

Overall the co-crystal structure indicates a conventional SH2-pTyr interaction, and the surface electrostatics of the SH2 domain support this observation, with a positively charged region encompassing the phosphotyrosine binding site, and a hydrophobic region at the +3 pocket (Fig 3D).

### Biochemical validation of the mode of p120RasGAP-p190RhoGAP interaction

**Fig 4:**
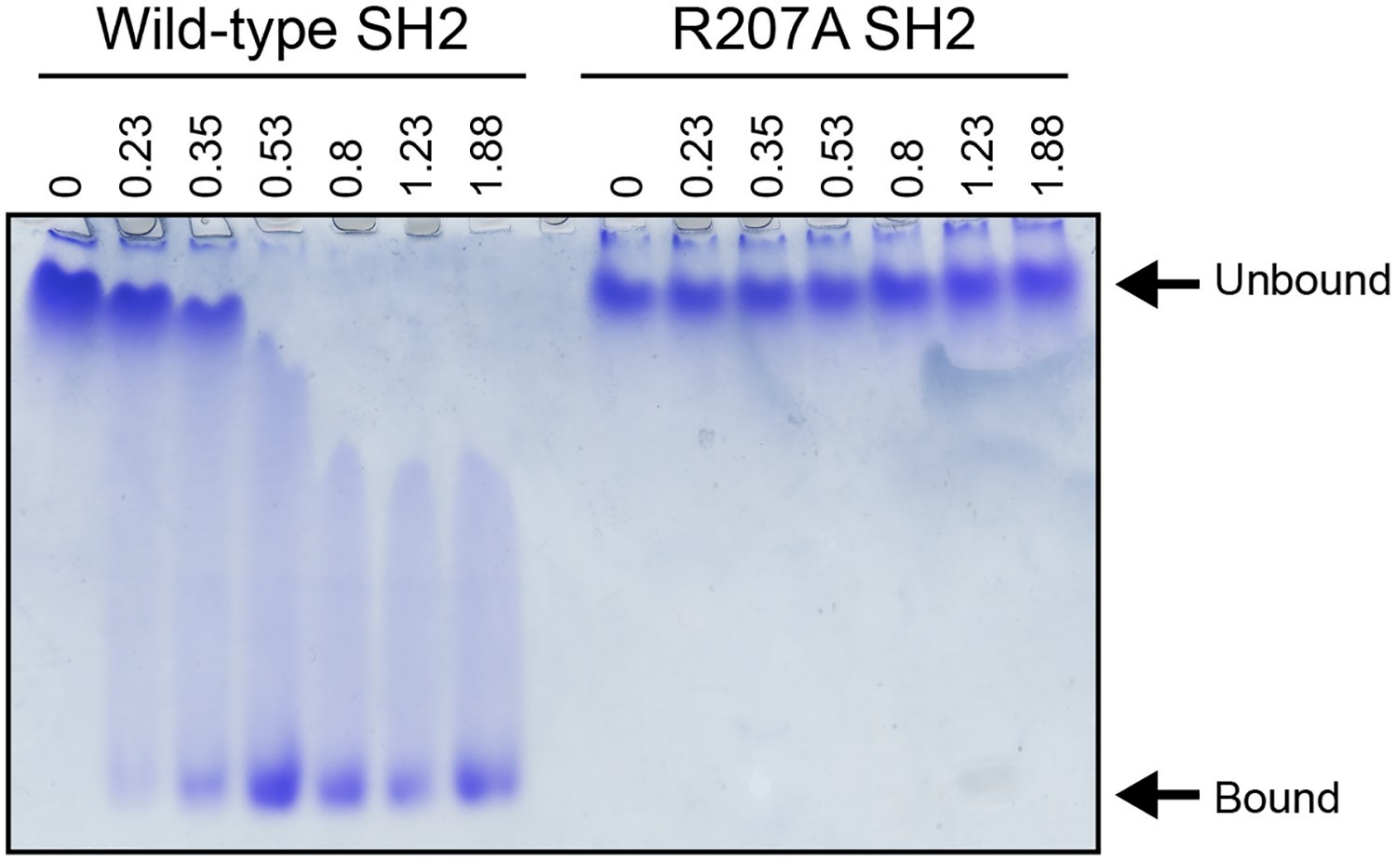
Native PAGE for p120RasGAP N-SH2 and p190RhoGAP pTyr-1105 peptide. Native PAGE illustrating the effect of increasing peptide concentrations on wild-type p120RasGAP N-SH2 (left) and R207A mutant p120RasGAP (right). Peptide:protein molar ratio indicated for each lane.

To validate the interaction that we observe crystallographically, we conducted native PAGE. The N-terminal SH2 domain of p120RasGAP slowly enters a native gel, however, addition of saturating concentrations of p190RhoGAP pTyr-1105 peptide result in a significant increase in its mobility in the gel (Fig 4 and S1 Fig). We hypothesize this to be due to an overall change in the surface charge of the complex compared to the apo SH2 domain [64]. The key conserved residue in almost all SH2 domains that is responsible for phosphotyrosine binding is the FLVR motif arginine, and its mutation to alanine is often used to generate a non-functional SH2 [56]. We introduced an R207A mutation into the N-terminal SH2 domain of p120RasGAP and find that addition of p190RhoGAP pTyr-1105 peptide to the mutant protein fails to shift (Fig 4). We interpret this to validate the importance of Arg-207 for the p120RasGAP N-SH2 interaction with p190RhoGAP pTyr-1105.

### Affinity measurements of the p120RasGAP-p190RhoGAP interaction

**Fig 5:**
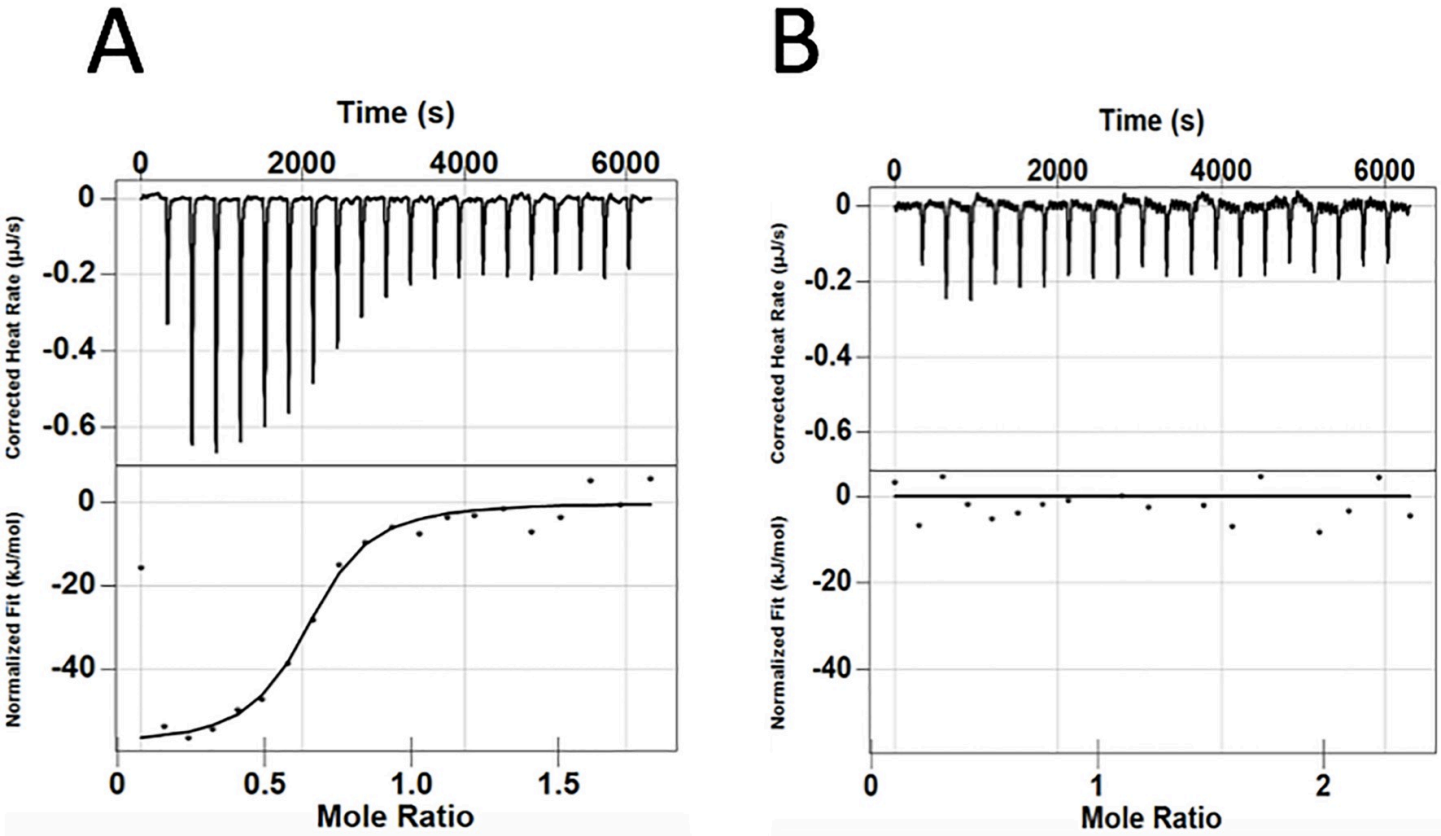
Isothermal titration calorimetry p190RhoGAP pTyr-1105 peptide titration into p120RasGAP N-SH2. **A)** p190RhoGAP pTyr-1105 peptide at 78.5 μM titrated into p120RasGAP N-SH2 at 15 μM. **B)** p190RhoGAP pTyr-1105 peptide at 120 μM titrated into R207A p120RasGAP N-SH2 at 15 μM.

We conducted isothermal titration calorimetry to assess the interaction between the N-terminal SH2 domain of p120RasGAP and the p190RhoGAP pTyr-1105 peptide. We injected the p190RhoGAP pTyr-1105 peptide at a concentration of 78.5 μM into N-SH2 at a concentration of 15 μM. Averaged over two independent experiments we observe a K_d_ of 0.3 ± 0.1 μM (Fig 5A). We do not observe measurable heat when we inject pTyr-1105 peptide at a concentration of 120 μM into R207A mutated N-SH2 at a concentration of 15 μM (Fig 5B).

## Conclusions

In this study, we determine the crystal structure of a key interaction between p190RhoGAP and p120RasGAP. For over two decades, extensive studies have been conducted on the interaction and consequences of binding [65], however, the molecular basis for the interaction has not, until now, been observed. The SH2 domains of p120RasGAP bind to pTyr-1105 of p190RhoGAP [3, 18, 19], and this is thought to be the most critical component of the interaction between the proteins–although frequently observed [22] there remains controversy about the importance of the nearby phosphorylation site in p190RhoGAP, pTyr-1087 [18]. Our structural study demonstrates that the interaction of the N-terminal SH2 domain with pTyr-1105 of p190RhoGAP is a canonical FLVR motif arginine mediated SH2-phosphotyrosine complex. Our affinity measurement for this interaction of 0.3±0.1 μM is similar to other SH2-pTyr interactions [61], and mutation of the FLVR motif arginine residue abolishes interaction. Based on Dali analysis, the structure is most similar to the C-terminal SH2 domain from phospholipase C-γ1 (Z-score 17.3, r.m.s.d. 1.4 Å over 97 residues) [66]. The N-terminal SH2 domain of p120RasGAP has a preference for proline at the pY+3 position [67], and the PLC-γ1 SH2-C prefers proline, valine and isoleucine [67]. Recently, however PLC-γ1 SH2-C was shown to be promiscuous in its binding specificity at this position [68] perhaps implying a similar diversity for other SH2 domains, including those of p120RasGAP. Overall, our crystal structures therefore demonstrate the mode of binding of a key interface between p190RhoGAP and p120RasGAP, and provide a molecular understanding of specificity determinants of the interaction.

## Supporting information

S1 Fig Uncropped gel for Fig 4. (TIF)

## References

[1] Barbacid M. ras genes. Annu Rev Biochem. 1987;56:779–827. Epub 1987/01/01. 10.1146/annurev.bi.56.070187.004023 . 3304147

[2] Hall A. Rho GTPases and the actin cytoskeleton. Science. 1998;279(5350):509–14. 10.1126/science.279.5350.509 . 9438836

[3] Bryant SS, Briggs S, Smithgall TE, Martin GA, McCormick F, Chang JH, et al Two SH2 domains of p120 Ras GTPase-activating protein bind synergistically to tyrosine phosphorylated p190 Rho GTPase-activating protein. J Biol Chem. 1995;270(30):17947–52. 10.1074/jbc.270.30.17947 . 7629101

[4] Wildenberg GA, Dohn MR, Carnahan RH, Davis MA, Lobdell NA, Settleman J, et al p120-catenin and p190RhoGAP regulate cell-cell adhesion by coordinating antagonism between Rac and Rho. Cell. 2006;127(5):1027–39. 10.1016/j.cell.2006.09.046 . 17129786

[5] Kulkarni SV, Gish G, van der Geer P, Henkemeyer M, Pawson T. Role of p120 Ras-GAP in directed cell movement. J Cell Biol. 2000;149(2):457–70. Epub 2000/04/18. 10.1083/jcb.149.2.457 . 10769036 PMC2175152

[6] van der Geer P, Henkemeyer M, Jacks T, Pawson T. Aberrant Ras regulation and reduced p190 tyrosine phosphorylation in cells lacking p120-Gap. Mol Cell Biol. 1997;17(4):1840–7. Epub 1997/04/01. 10.1128/mcb.17.4.1840 . 9121432 PMC232031

[7] Chang JH, Gill S, Settleman J, Parsons SJ. c-Src regulates the simultaneous rearrangement of actin cytoskeleton, p190RhoGAP, and p120RasGAP following epidermal growth factor stimulation. J Cell Biol. 1995;130(2):355–68. 10.1083/jcb.130.2.355 . 7542246 PMC2199934

[8] Sfakianos MK, Eisman A, Gourley SL, Bradley WD, Scheetz AJ, Settleman J, et al Inhibition of Rho via Arg and p190RhoGAP in the postnatal mouse hippocampus regulates dendritic spine maturation, synapse and dendrite stability, and behavior. J Neurosci. 2007;27(41):10982–92. 10.1523/JNEUROSCI.0793-07.2007 . 17928439 PMC6672862

[9] Bradley WD, Hernandez SE, Settleman J, Koleske AJ. Integrin signaling through Arg activates p190RhoGAP by promoting its binding to p120RasGAP and recruitment to the membrane. Mol Biol Cell. 2006;17(11):4827–36. 10.1091/mbc.E06-02-0132 . 16971514 PMC1635390

[10] Arthur WT, Burridge K. RhoA inactivation by p190RhoGAP regulates cell spreading and migration by promoting membrane protrusion and polarity. Mol Biol Cell. 2001;12(9):2711–20. 10.1091/mbc.12.9.2711 . 11553710 PMC59706

[11] Ridley AJ, Self AJ, Kasmi F, Paterson HF, Hall A, Marshall CJ, et al rho family GTPase activating proteins p190, bcr and rhoGAP show distinct specificities in vitro and in vivo. EMBO J. 1993;12(13):5151–60. . 8262058 10.1002/j.1460-2075.1993.tb06210.x PMC413777

[12] Stiegler AL, Boggon TJ. PseudoGTPase domains in p190RhoGAP proteins: a mini-review. Biochem Soc Trans. 2018;46(6):1713–20. Epub 2018/12/06. 10.1042/BST20180481 . 30514771 PMC6501215

[13] Stiegler AL, Boggon TJ. The N-terminal GTPase domain of p190RhoGAP proteins is a pseudoGTPase. Structure. 2018;26(11):1451–61. 10.1016/j.str.2018.07.015 . 30174148 PMC6249675

[14] Stiegler AL, Boggon TJ. p190RhoGAP proteins contain pseudoGTPase domains. Nature communications. 2017;8(1):506 Epub 2017/09/13. 10.1038/s41467-017-00483-x . 28894085 PMC5593906

[15] Burbelo PD, Miyamoto S, Utani A, Brill S, Yamada KM, Hall A, et al p190-B, a new member of the Rho GAP family, and Rho are induced to cluster after integrin cross-linking. J Biol Chem. 1995;270(52):30919–26. 10.1074/jbc.270.52.30919 . 8537347

[16] LeClerc S, Palaniswami R, Xie BX, Govindan MV. Molecular cloning and characterization of a factor that binds the human glucocorticoid receptor gene and represses its expression. J Biol Chem. 1991;266(26):17333–40. . 1894621

[17] Settleman J, Narasimhan V, Foster LC, Weinberg RA. Molecular cloning of cDNAs encoding the GAP-associated protein p190: implications for a signaling pathway from ras to the nucleus. Cell. 1992;69(3):539–49. 10.1016/0092-8674(92)90454-k . 1581965

[18] Roof RW, Haskell MD, Dukes BD, Sherman N, Kinter M, Parsons SJ. Phosphotyrosine (p-Tyr)-dependent and -independent mechanisms of p190 RhoGAP-p120 RasGAP interaction: Tyr 1105 of p190, a substrate for c-Src, is the sole p-Tyr mediator of complex formation. Mol Cell Biol. 1998;18(12):7052–63. 10.1128/mcb.18.12.7052 . 9819392 PMC109287

[19] Haskell MD, Nickles AL, Agati JM, Su L, Dukes BD, Parsons SJ. Phosphorylation of p190 on Tyr1105 by c-Src is necessary but not sufficient for EGF-induced actin disassembly in C3H10T1/2 fibroblasts. J Cell Sci. 2001;114(Pt 9):1699–708. . 11309200 10.1242/jcs.114.9.1699

[20] Hu KQ, Settleman J. Tandem SH2 binding sites mediate the RasGAP-RhoGAP interaction: a conformational mechanism for SH3 domain regulation. EMBO J. 1997;16(3):473–83. 10.1093/emboj/16.3.473 . 9034330 PMC1169651

[21] Hernandez SE, Settleman J, Koleske AJ. Adhesion-dependent regulation of p190RhoGAP in the developing brain by the Abl-related gene tyrosine kinase. Curr Biol. 2004;14(8):691–6. 10.1016/j.cub.2004.03.062 . 15084284

[22] Hornbeck PV, Zhang B, Murray B, Kornhauser JM, Latham V, Skrzypek E. PhosphoSitePlus, 2014: mutations, PTMs and recalibrations. Nucleic Acids Res. 2015;43(Database issue):D512–20. 10.1093/nar/gku1267 . 25514926 PMC4383998

[23] Pamonsinlapatham P, Hadj-Slimane R, Lepelletier Y, Allain B, Toccafondi M, Garbay C, et al p120-Ras GTPase activating protein (RasGAP): a multi-interacting protein in downstream signaling. Biochimie. 2009;91(3):320–8. 10.1016/j.biochi.2008.10.010 . 19022332

[24] McCormick F, Adari H, Trahey M, Halenbeck R, Koths K, Martin GA, et al Interaction of ras p21 proteins with GTPase activating protein. Cold Spring Harbor symposia on quantitative biology. 1988;53 Pt 2:849–54. Epub 1988/01/01. 10.1101/sqb.1988.053.01.097 . 2855502

[25] Adari H, Lowy DR, Willumsen BM, Der CJ, McCormick F. Guanosine triphosphatase activating protein (GAP) interacts with the p21 ras effector binding domain. Science. 1988;240(4851):518–21. Epub 1988/04/22. 10.1126/science.2833817 . 2833817

[26] Wang J, Tian X, Han R, Zhang X, Wang X, Shen H, et al Downregulation of miR-486-5p contributes to tumor progression and metastasis by targeting protumorigenic ARHGAP5 in lung cancer. Oncogene. 2014;33(9):1181–9. Epub 2013/03/12. 10.1038/onc.2013.42 . 23474761 PMC3883922

[27] Lawrence MS, Stojanov P, Mermel CH, Robinson JT, Garraway LA, Golub TR, et al Discovery and saturation analysis of cancer genes across 21 tumour types. Nature. 2014;505(7484):495–501. 10.1038/nature12912 . 24390350 PMC4048962

[28] Fang Y, Zhu X, Wang J, Li N, Li D, Sakib N, et al MiR-744 functions as a proto-oncogene in nasopharyngeal carcinoma progression and metastasis via transcriptional control of ARHGAP5. Oncotarget. 2015;6(15):13164–75. Epub 2015/05/12. 10.18632/oncotarget.3754 . 25961434 PMC4537006

[29] Sordella R, Classon M, Hu KQ, Matheson SF, Brouns MR, Fine B, et al Modulation of CREB activity by the Rho GTPase regulates cell and organism size during mouse embryonic development. Dev Cell. 2002;2(5):553–65. 10.1016/s1534-5807(02)00162-4 . 12015964

[30] Su L, Pertz O, Mikawa M, Hahn K, Parsons SJ. p190RhoGAP negatively regulates Rho activity at the cleavage furrow of mitotic cells. Exp Cell Res. 2009;315(8):1347–59. Epub 2009/03/04. 10.1016/j.yexcr.2009.02.014 . 19254711 PMC2731427

[31] Moran MF, Polakis P, McCormick F, Pawson T, Ellis C. Protein-tyrosine kinases regulate the phosphorylation, protein interactions, subcellular distribution, and activity of p21ras GTPase-activating protein. Mol Cell Biol. 1991;11(4):1804–12. Epub 1991/04/01. 10.1128/mcb.11.4.1804 . 2005883 PMC359849

[32] Bernards A, Settleman J. GAP control: regulating the regulators of small GTPases. Trends Cell Biol. 2004;14(7):377–85. Epub 2004/07/13. 10.1016/j.tcb.2004.05.003 . 15246431

[33] Bos JL, Rehmann H, Wittinghofer A. GEFs and GAPs: critical elements in the control of small G proteins. Cell. 2007;129(5):865–77. 10.1016/j.cell.2007.05.018 . 17540168

[34] Trahey M, McCormick F. A cytoplasmic protein stimulates normal N-ras p21 GTPase, but does not affect oncogenic mutants. Science. 1987;238(4826):542–5. Epub 1987/10/23. 10.1126/science.2821624 . 2821624

[35] Campbell JD, Alexandrov A, Kim J, Wala J, Berger AH, Pedamallu CS, et al Distinct patterns of somatic genome alterations in lung adenocarcinomas and squamous cell carcinomas. Nat Genet. 2016;48(6):607–16. Epub 2016/05/10. 10.1038/ng.3564 . 27158780 PMC4884143

[36] Chan PC, Chen HC. p120RasGAP-mediated activation of c-Src is critical for oncogenic Ras to induce tumor invasion. Cancer Res. 2012;72(9):2405–15. Epub 2012/03/14. 10.1158/0008-5472.CAN-11-3078 . 22411953

[37] Berndt SI, Wang Z, Yeager M, Alavanja MC, Albanes D, Amundadottir L, et al Two susceptibility loci identified for prostate cancer aggressiveness. Nature communications. 2015;6:6889 Epub 2015/05/06. 10.1038/ncomms7889 . 25939597 PMC4422072

[38] Antoine-Bertrand J, Duquette PM, Alchini R, Kennedy TE, Fournier AE, Lamarche-Vane N. p120RasGAP Protein Mediates Netrin-1 Protein-induced Cortical Axon Outgrowth and Guidance. J Biol Chem. 2016;291(9):4589–602. Epub 2015/12/30. 10.1074/jbc.M115.674846 . 26710849 PMC4813483

[39] Wang DZ, Nur EKMS, Tikoo A, Montague W, Maruta H. The GTPase and Rho GAP domains of p190, a tumor suppressor protein that binds the M(r) 120,000 Ras GAP, independently function as anti-Ras tumor suppressors. Cancer Res. 1997;57(12):2478–84. . 9192829

[40] Boon LM, Mulliken JB, Vikkula M. RASA1: variable phenotype with capillary and arteriovenous malformations. Curr Opin Genet Dev. 2005;15(3):265–9. 10.1016/j.gde.2005.03.004 . 15917201

[41] Eerola I, Boon LM, Mulliken JB, Burrows PE, Dompmartin A, Watanabe S, et al Capillary malformation-arteriovenous malformation, a new clinical and genetic disorder caused by RASA1 mutations. Am J Hum Genet. 2003;73(6):1240–9. Epub 2003/11/26. 10.1086/379793 . 14639529 PMC1180390

[42] Revencu N, Boon LM, Mulliken JB, Enjolras O, Cordisco MR, Burrows PE, et al Parkes Weber syndrome, vein of Galen aneurysmal malformation, and other fast-flow vascular anomalies are caused by RASA1 mutations. Hum Mutat. 2008;29(7):959–65. Epub 2008/05/01. 10.1002/humu.20746 . 18446851

[43] Revencu N, Boon LM, Mendola A, Cordisco MR, Dubois J, Clapuyt P, et al RASA1 mutations and associated phenotypes in 68 families with capillary malformation-arteriovenous malformation. Hum Mutat. 2013;34(12):1632–41. Epub 2013/09/17. 10.1002/humu.22431 . 24038909

[44] Otwinowski Z, Minor W. Processing of X-ray diffraction data collected in oscillation mode In: Carter CW, Sweet RM, editors. Methods in Enzymology. 276, Part A. San Diego: Academic Press (New York); 1997 p. 307–26. 10.1016/S0076-6879(97)76066-X 27754618

[45] Adams PD, Afonine PV, Bunkoczi G, Chen VB, Davis IW, Echols N, et al PHENIX: a comprehensive Python-based system for macromolecular structure solution. Acta Crystallogr D Biol Crystallogr. 2010;66(Pt 2):213–21. 10.1107/S0907444909052925 . 20124702 PMC2815670

[46] McCoy AJ, Grosse-Kunstleve RW, Adams PD, Winn MD, Storoni LC, Read RJ. Phaser crystallographic software. Journal of applied crystallography. 2007;40(Pt 4):658–74. 10.1107/S0021889807021206 . 19461840 PMC2483472

[47] Terwilliger TC, Grosse-Kunstleve RW, Afonine PV, Moriarty NW, Zwart PH, Hung LW, et al Iterative model building, structure refinement and density modification with the PHENIX AutoBuild wizard. Acta Crystallogr D Biol Crystallogr. 2008;64(Pt 1):61–9. Epub 2007/12/21. 10.1107/S090744490705024X . 18094468 PMC2394820

[48] Lebedev AA, Isupov MN. Space-group and origin ambiguity in macromolecular structures with pseudo-symmetry and its treatment with the program Zanuda. Acta Crystallogr D Biol Crystallogr. 2014;70(Pt 9):2430–43. 10.1107/S1399004714014795 . 25195756

[49] Emsley P, Lohkamp B, Scott WG, Cowtan K. Features and development of Coot. Acta Crystallogr D Biol Crystallogr. 2010;66(Pt 4):486–501. 10.1107/S0907444910007493 . 20383002 PMC2852313

[50] Chen VB, Arendall WB 3rd, Headd JJ, Keedy DA, Immormino RM, Kapral GJ, et al MolProbity: all-atom structure validation for macromolecular crystallography. Acta Crystallogr D Biol Crystallogr. 2010;66(Pt 1):12–21. 10.1107/S0907444909042073 . 20057044 PMC2803126

[51] McNicholas S, Potterton E, Wilson KS, Noble ME. Presenting your structures: the CCP4mg molecular-graphics software. Acta Crystallogr D Biol Crystallogr. 2011;67(Pt 4):386–94. 10.1107/S0907444911007281 . 21460457 PMC3069754

[52] Krissinel E, Henrick K. Inference of macromolecular assemblies from crystalline state. J Mol Biol. 2007;372(3):774–97. 10.1016/j.jmb.2007.05.022 . 17681537

[53] Morin A, Eisenbraun B, Key J, Sanschagrin PC, Timony MA, Ottaviano M, et al Cutting edge: Collaboration gets the most out of software. eLife. 2013;2:e01456 10.7554/eLife.01456 24040512 PMC3771563

[54] Zhang ZY, Maclean D, Thieme-Sefler AM, Roeske RW, Dixon JE. A continuous spectrophotometric and fluorimetric assay for protein tyrosine phosphatase using phosphotyrosine-containing peptides. Anal Biochem. 1993;211(1):7–15. Epub 1993/05/15. 10.1006/abio.1993.1224 . 7686722

[55] Waksman G, Kominos D, Robertson SC, Pant N, Baltimore D, Birge RB, et al Crystal structure of the phosphotyrosine recognition domain SH2 of v-src complexed with tyrosine-phosphorylated peptides. Nature. 1992;358(6388):646–53. 10.1038/358646a0 . 1379696

[56] Waksman G, Kuriyan J. Structure and specificity of the SH2 domain. Cell. 2004;116(2 Suppl):S45–8, 3 p following S8. 10.1016/s0092-8674(04)00043-1 . 15055581

[57] Eck MJ, Shoelson SE, Harrison SC. Recognition of a high-affinity phosphotyrosyl peptide by the Src homology-2 domain of p56^*lck*^. Nature. 1993;362:87–91. 10.1038/362087a0 7680435

[58] Overduin M, Rios CB, Mayer BJ, Baltimore D, Cowburn D. Three-Dimensional Solution Structure of the src Homology 2 Domain of c-abl. Cell. 1992;70:697–704. 10.1016/0092-8674(92)90437-h 1505033

[59] Booker GW, Breeze AL, Downing AK, Panayotou G, Gout I, Waterfield MD, et al Structure of SH2 domain of the p85a subunit of phosphatidylinositol-3-OH kinase. Nature. 1992;358:684–7. 10.1038/358684a0 1323062

[60] Songyang Z, Shoelson SE, McGlade J, Olivier P, Pawson T, Bustelo XR, et al Specific motifs recognized by the SH2 domains of Csk 3BP2, fps/fes, Grb-2, HCP, SHC, Syk and Vav. Mol Cell Biol. 1994;14:2777–85. 10.1128/mcb.14.4.2777 7511210 PMC358643

[61] Huang H, Li L, Wu C, Schibli D, Colwill K, Ma S, et al Defining the specificity space of the human SRC homology 2 domain. Mol Cell Proteomics. 2008;7(4):768–84. Epub 2007/10/25. 10.1074/mcp.M700312-MCP200 . 17956856

[62] Songyang Z, Shoelson SE, Chaudhuri M, Gish G, Pawson T, Haser WG, et al SH2 domains recognize specific phosphopeptide sequences. Cell. 1993;72(5):767–78. Epub 1993/03/12. 10.1016/0092-8674(93)90404-e . 7680959

[63] Hidaka M, Homma Y, Takenawa T. Highly conserved eight amino acid sequence in SH2 is important for recognition of phosphotyrosine site. Biochem Biophys Res Commun. 1991;180(3):1490–7. Epub 1991/11/14. 10.1016/s0006-291x(05)81364-6 . 1719984

[64] Boggon TJ, Shapiro L. Screening for phasing atoms in protein crystallography. Structure. 2000;8(7):R143–9. 10.1016/s0969-2126(00)00168-4 ot required. 10903954

[65] Heraud C, Pinault M, Lagree V, Moreau V. p190RhoGAPs, the ARHGAP35- and ARHGAP5-Encoded Proteins, in Health and Disease. Cells. 2019;8(4). Epub 2019/04/25. 10.3390/cells8040351 . 31013840 PMC6523970

[66] Holm L, Rosenstrom P. Dali server: conservation mapping in 3D. Nucleic Acids Res. 2010;38(Web Server issue):W545–9. 10.1093/nar/gkq366 . 20457744 PMC2896194

[67] Liu BA, Engelmann BW, Nash PD. The language of SH2 domain interactions defines phosphotyrosine-mediated signal transduction. FEBS Lett. 2012;586(17):2597–605. Epub 2012/05/10. 10.1016/j.febslet.2012.04.054 . 22569091

[68] McKercher MA, Guan X, Tan Z, Wuttke DS. Multimodal Recognition of Diverse Peptides by the C-Terminal SH2 Domain of Phospholipase C-gamma1 Protein. Biochemistry. 2017;56(16):2225–37. Epub 2017/04/05. 10.1021/acs.biochem.7b00023 . 28376302

[69] Meyer PA, Socias S, Key J, Ransey E, Tjon EC, Buschiazzo A, et al Data publication with the structural biology data grid supports live analysis. Nature communications. 2016;7:10882 10.1038/ncomms10882 . 26947396 PMC4786681

