# Correction: Crystal structures of p120RasGAP N-terminal SH2 domain in its apo form and in complex with a p190RhoGAP phosphotyrosine peptide

**DOI:** 10.1371/journal.pone.0229627

**Published:** 2020-02-20

**Authors:** Rachel Jaber Chehayeb, Amy L. Stiegler, Titus J. Boggon

In the Funding section, the grant number from the funder National Institutes of Health is listed incorrectly. The correct grant number is: R01GM102262.
