# Supplementary figures and images for: Crystal structures of p120RasGAP N-terminal SH2 domain in its apo form and in complex with a p190RhoGAP phosphotyrosine peptide
Source: PLoS One. 2019 Dec 31;14(12):e0226113. doi: 10.1371/journal.pone.0226113 (PMC6938330; doi:10.1371/journal.pone.0226113)

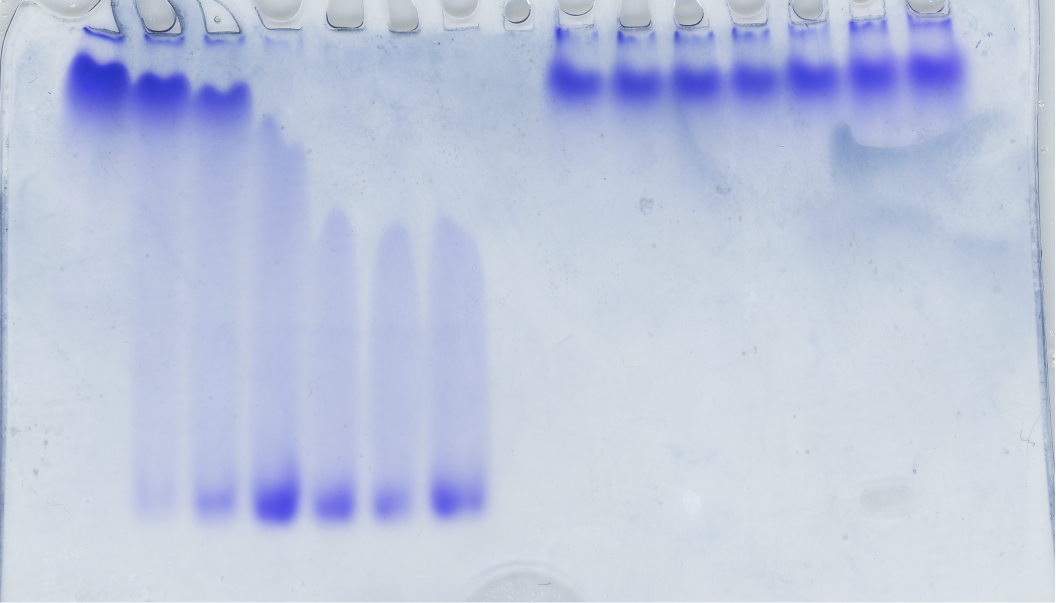

Supplement: S1 Fig — (TIF) [file pone.0226113.s001.tif]
